# What makes a specialised emergency plan effective?—A case study of railway operation in 33 prefectural cities in China

**DOI:** 10.1371/journal.pone.0327007

**Published:** 2025-07-24

**Authors:** Cuixi Li, Yibao Wang, Wen Qing, Chong Li

**Affiliations:** School of Public Policy and Management (School of Emergency Management), China University of Mining and Technology, Xuzhou, China; Central South University, CHINA

## Abstract

Emergency response plans play a crucial role in mitigating safety risks associated with urban rail transport operations and effectively responding to emergencies in underground spaces. This study evaluates and analyzes the emergency response plans for rail transit operations in 33 cities in China, focusing on the consistency and completeness of the plans. The evaluation index system for Urban Rail Transit Operation Emergency Plans (URTOEPs) consists of 9 primary variables and 42 secondary variables, constructed based on the text analysis and the Policy Model Consistency (PMC) index model. It fills the gap of more fragmented analysis and less holistic quantitative assessment in the research of established urban rail transit emergency plans. The results indicate that URTOEPs are well-designed, but there is still room for improvement. The mean PMC index value for 33 URTOEPs is 7.83, which reflects a good grade overall. Of these, 11 URTOEPs achieved an excellent grade while 22 were rated as good. Meanwhile, based on the mean PMC index of first-level variables and the PMC-surface diagrams of six representative plans, the emergency plans need significant improvement in restoration and reconstruction, plan application, and preventive preparedness. This study enriches the research related to text quality assessment of emergency plans, identifies structural deficiencies in the plan system through quantitative assessment, and provides empirical references for the optimization of urban rail transit emergency plans and the formulation of policies related to urban risk management.

## 1 Introduction

With the rapid development of the global economy, an increasing number of countries have begun to accelerate the process of urbanization. Due to the rapid expansion of urban capacity and growing population density, some urban functions have gradually been relocated to underground spaces. Consequently, potential safety risks have started to accumulate rapidly in urban underground areas. In recent years, rail transport has gained significant momentum as a crucial functional component of urban underground spaces in both domestic and international city construction projects [1,2]. As of the end of 2023, urban rail systems spanning over 43,400.40 km were operational in 563 cities across 79 countries and territories [3]. However, a series of extreme meteorological disasters combined with underground infrastructure have exposed un-precedented safety risks for urban rail transit [4], resulting in significant property damage and casualties. Notable incidents include the Zhengzhou underground flood in China on ‘7-20’ [5], train collisions and derailments in New York City, Seoul, South Korea, and Odisha, India. These events underscore the urgent need to enhance emergency management capabilities within urban rail transportation. The quality of Urban Rail Transit Emergency Operation Plans (URTOEPs), as a crucial component for prevention and preparedness within the emergency management lifecycle [6–8], directly impacts the actual effectiveness of emergency response.

The State Council of China issued the National Emergency Response System Plan for the 14th Five-Year Plan Period on December 30, 2021, emphasizing the imperative to enhance emergency response plan preparedness, expedite plan preparation and re-vision processes, and accelerate plan revision. The higher the level, the more comprehensive and informative the emergency response plan becomes; conversely, at lower levels, practicality takes precedence in shaping the emergency response plan. Nevertheless, there exist deficiencies in terms of practicality and applicability within emergency plans, impeding their potential to be fully effective during emergency scenarios. The emergency plan for rail transport at the municipal level plays a crucial role as a key link between the provincial level and operating units. It is essential that this plan not only be well-structured, instructive, and operational but also consider effective co-ordination and interaction among multiple departments and units horizontally. Failure to have a comprehensive emergency response plan in place for urban rail transport operations during emergencies such as train derailments, fires, or terrorist attacks can lead to significant casualties, property losses, and even societal instability. For instance, during the 2021 rainstorm event in Zhengzhou, Henan province of China, when underground line 5 experienced widespread flooding, there was a lack of effective command leadership and timely implementation of emergency response measures. It took two hours before the decision to halt operations was made without activating any emergency response procedures throughout the process. Consequently, the existing emergency response plan proved ineffective as it failed to provide efficient guidance. Therefore, urgent attention from both academic and practical communities is required to enhance the quality of Urban Rail Transport Emergency Preparedness (URTOEPs) documents.

In previous studies, the focus of urban rail transit emergency management has primarily revolved around practical activities at various levels, including the establishment of emergency facilities for urban rail transit [9], response to emergencies [10], decision-making during emergencies [11,12], evacuation procedures during emergencies [13,14], and rescue operations in emergency situations [15]. Although the high-quality research on Urban Rail Transit Operation Emergency Plans (URTOEPs) has garnered attention from the academic community, there still exist certain issues in current research. On one hand, there is a dearth of comprehensive quantitative analyses on URTOEPs in China. On the other hand, the specificity of emergency plans at the municipal level has been overlooked. What are the overall textual characteristics of URTOEPs in China? Are there significant disparities among different cities? How about the completeness of individual emergency plans? To address these three inquiries, this study will conduct a comparative quantitative analysis on 33 URTOEPs.

Specifically, this paper firstly applies the PMC index model to construct an evaluation index system for emergency response plans in urban rail transport operations. Secondly, it quantitatively calculates and evaluates the PMC index of 33 emergency plans for urban rail transport operation emergencies. Then, it draws PMC surface diagrams of representative emergency plans. Finally, based on the evaluation results, it analyzes the overall characteristics and component characteristics of the text quality of the emergency plans, leading to research conclusions and policy recommendations. Therefore, the main contributions of this paper are twofold: (1) In contrast to previous relevant studies, this research focuses on evaluating the text of emergency response plans for urban rail transit operation incidents, which are widely utilized in urban safety management practices and specifically designed for non-routine situations. This selection provides a micro-sample for examining the establishment of China’s emergency management system. (2) Diverging from prior studies that primarily discussed policy perspectives such as emergency plan preparation and policy revision agenda, this research emphasizes a technical standpoint and employs quantitative methods to explore the standardization of fundamental content within special emergency response plans. This serves as a complementary aspect to existing research on evaluation indicators for emergency response plans while also providing data support for optimizing the construction and dynamic management of these specialized plans.

The paper’s basic framework is structured as follows: the second section presents a comprehensive review of the relevant literature. The third part elucidates the data sources and outlines the process of model construction. The fourth part conducts an analysis of the quantitative evaluation results. Lastly, in the fifth part, a summary of findings is provided along with further discussion on policy recommendations and fu-ture research prospects.

## 2 Literature review

### 2.1 Research on emergency response plans for urban rail transit

The emergency plan is a comprehensive administrative document designed to promptly and efficiently address emergencies or disasters [7]. As a tangible outcome of preparedness activities throughout the emergency management life cycle, it delineates the requirements for emergency response in exceptional scenarios and outlines the program for addressing the incident [16]. The origins of emergency planning can be traced back to 1974, when the Health and Safety Commission of the United Kingdom proposed an emergency plan in response to the explosion at the Flix-borough chemical plant [17]. Developing a high-quality emergency planning system has become an essential component of effective emergency management. For instance, the US federal government has established a range of comprehensive plans for responding to emergencies, including the National Response Plan in 1968, the Federal Response Plan in 1999, the National Response Plan in 2004, and the National Response Framework in 2008 [7]. The emergency planning system framework was formulated by France in 2006, while Japan revised its basic plan for disaster prevention in 2022 [18]. China established an emergency management system centered on the “one case and three systems” (emergency plan, emergency management system, emergency management mechanism, and emergency management legal system) following the SARS epidemic in 2003. The emergency plan holds a crucial position in the establishment of the emergency management system, serving as its leader and acting as the starting point for implementing the “one case, three systems” approach [19].

**Fig 1:**
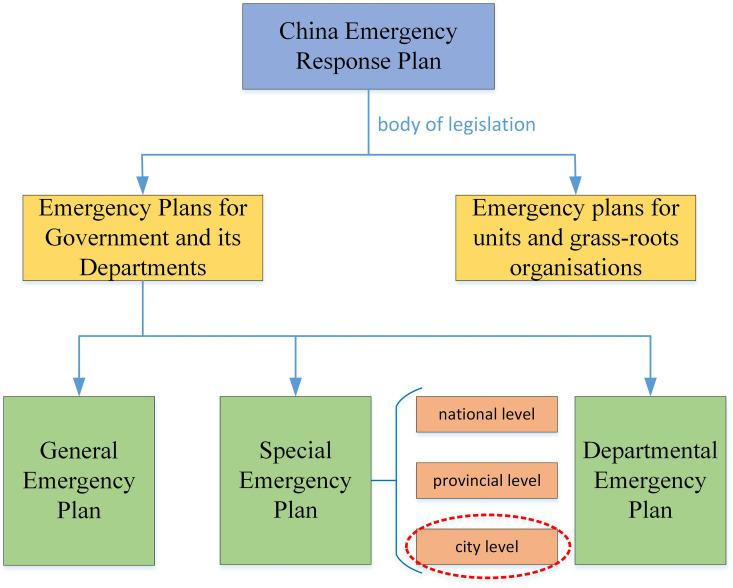
Classification of emergency response plans for emergencies in China.

The emergency response plans for emergencies in China can be categorized into two main types based on the responsible entities: government and its departments’ emergency response plans, and units and grass-roots organizations’ emergency response plans. Among these, the government and its departmental emergency response plans encompass general, special, and department-specific ones (refer to Fig 1). The special emergency plan is a pre-formulated program by the government, involving multiple departments and their respective responsibilities, aimed at effectively responding to specific types of emergencies or carrying out important tasks such as protecting critical targets, ensuring the safety of major events, and implementing emergency measures. URTOEPs belong to the special plan in China’s emergency response plan system. These plans primarily offer specific and detailed strategies and measures for addressing emergencies in the field of urban rail transit operation. URTOEPs not only constitute a crucial component of the national emergency response plan system but also provide significant support for ensuring the safety management of urban rail transit operation.

Compared to ground systems, urban rail transport systems are characterized by their relatively enclosed nature, high population density, and challenges in terms of evacuation and rescue. These factors contribute to the increased complexity and un-certainty of disaster risk. Additionally, unclear emergency management responsibilities and a lack of clear division of labor further hinder effective emergency management in urban rail transit. Therefore, the key focus should be on enhancing emergency response plans as well as improving organizational capabilities for emergencies. From the perspective of research themes, current studies on urban rail transit emergency response plans encompass enhancing the enforceability of emergency response plans through management methods [20], modeling and analyzing the emergency response process [21], investigating emergency evacuation based on emergency response plans [22], and constructing models for emergency rescue [23,24]. Although some scholars have also made attempts to establish an assessment index system for metro emergency plans [25], the current research on quality evaluation of emergency plans for urban rail transport emergencies is still in its nascent stage, with relatively limited direct literature available.

Overall, these studies have established a solid theoretical and methodological foundation for enhancing and advancing urban rail transport emergency plans. However, specific studies have overlooked the comparative analysis of the textual quality of these plans, as well as neglected to explore their optimization in terms of acceptability, flexibility, and specificity. In other words, this also prompts us to consider the assessment of the quality of urban rail transport emergency plans.

### 2.2 Research on policy evaluation based on the PMC index model

The Policy Modelling Consistency Index (PMC) model was proposed by Mario Arturo Ruiz Estrada [26], who applied the Omnia Mobilis hypothesis in a multidisciplinary approach to policy modelling. Under the assumption that ‘everything is moving’, he argued for the inclusion of a comprehensive set of variables and emphasized the importance of not overlooking any relevant factors [26]. Building upon this concept, Mario Arturo Ruiz Estrada introduced the PMC-Index model to evaluate policy coherence through a multidimensional system of assessment indicators. In comparison with other methods for assessing policy quality such as fuzzy hierarchical analysis, object-element analysis, hesitant fuzzy linguistic set, and network hierarchy-entropy weight analysis, the advantage of the PMC-Index model lies in its origin from within the policy itself, rendering it more objective. Additionally, it effectively reduces costs associated with policy analysis [27].

PMC models are extensively utilized in policy evaluation, encompassing economic policy [28,29], ecological policy [30,31], land policy [32,33]and other domains. Its associated research can be primarily categorized into two aspects: assessment of policy content and investigation of the effects on formulation and implementation. In recent years, the index model has also been gradually employed in the realm of security management policy evaluation. Although current research outcomes remain somewhat limited and lack systematicity, it offers a fresh perspective and stimulates profound exploration into emergency management policies. Examples include policies for fire safety education [27], energy safety [34], disaster recovery [35], and critical incident response [36]. Additionally, several scholars have applied the PMC model to assess the quality of emergency plans. For instance, Yi Xu et al [37] utilized the PMC model to quantitatively evaluate and analyze provincial public health emergency plans in China. Jian Li et al [38] assessed the overall emergency plan for emergencies in the Beijing-Tianjin-Hebei region by constructing a PMC index model, which provided suggestions for plan optimization.

To summarize, the research on emergency preparedness of existing urban rail transit presents the research characteristics of “three more and three less”. From the research perspective, there are more fragmented analysis and less systematic research. Existing research focuses on a single dimension of the emergency plan, such as technical standards, response process or departmental responsibilities, and lacks systematic integration of multidimensional perspectives. In terms of research content, there are more technical application studies and fewer policy evaluation studies. Research on urban rail transit emergency response plans focuses on the digital construction of emergency response plans, the design of emergency response plan calculation platforms, and the construction of emergency response plan management systems, etc., while research on text quality assessment based on quantitative analysis tools is still in the exploratory stage. In terms of research methodology, there is more qualitative interpretation and less quantitative assessment. Existing results mostly adopt qualitative analysis methods, focusing on the framework for the preparation of emergency plans, the emergency management mechanism and the summarization of case experiences. In fact, emergency response plans for unexpected events encompass the entire process of emergency management, including monitoring and warning, prevention and preparedness, emergency response, and recovery and reconstruction. These plans are characterized by their systematic approach and comprehensive coverage. As a policy evaluation tool, the PMC model enables a thorough and systematic analysis of each aspect of the emergency response plan to assess its strengths, weaknesses, and impacts. However, no scholars have yet applied this model to assess the policy quality of urban rail transit emergency plans. Therefore, this study evaluates the textual consistency and coherence of China’s URTOEPs based on the PMC index model while proposing optimization strategies to address any deficiencies in the text.

## 3 Materials and methods

### 3.1 Data sources and research sample

According to the Annual Statistics and Analysis Report on Urban Rail Transit 2022, released by the China Urban Rail Transit Association in March 2023, a total of 55 cities in China had commenced operations of urban rail transit lines by the end of 2022. The emergency plans from these cities were collected as research samples through official websites of municipal governments, the Department of Emergency Management’s official website, or emergency management plans featured in news topics on the Chinese government website. However, six of these cities lack independent emergency plans specifically tailored for urban rail transit operation emergencies. Instead, they rely on general rail transit regulations, safety management measures for rail transit operations, emergency plans for road traffic emergencies or comprehensive transport emergency plans. 16 cities were unable to obtain their official public sources’ emergency plans.

Therefore, the emergency response plans for rail transport operation emergencies in 33 cities were ultimately obtained as the sample pool for the study (refer to Table 1). From the perspective of regional distribution, the sample presents significant unbalanced characteristics: 18 samples in the eastern region, reflecting the strength of its policy supply as a high-density urban rail transit operation region; 7 samples in the central and western regions, and 1 sample in the northeast region, presenting a gradient difference between the east and the west, which is more consistent with the level of China’s regional economic development, the level of development of urban underground space and the scale of rail transit operation. From the policy time dimension, the sample covers the period from April 2014 to July 2023, with significant differences in the annual release frequency, of which 2020 and 2021 are the peak periods of policy release, which is mutually corroborated with the development trajectory of China’s urban rail transit scale expansion.

**Table 1:** Summary of emergency plan evaluation samples.

Sample number	Name of the emergency plan	Publication date	Length of lines in operation in 2022 (kilometers)	Region
P1	Emergency Response Plan for Emergencies in Shanghai Railway Transportation Operations (2020 Edition)	December 2020	936.17	Eastern
P2	Emergency Response Plan for Beijing Railway Traffic Operation Emergencies	January 2017	868.37	Eastern
P3	Emergency Response Plan for Guangzhou Urban Railway Transportation Operation Emergencies	November 2020	621.58	Eastern
P4	Emergency Response Plan for Emergencies in Urban Rail Transit Operations in Hangzhou (Revised in 2019)	January 2020	516	Eastern
P5	Nanjing Emergency Response Plan for Urban Rail Transit Operation Emergencies	December 2020	465.77	Eastern
P6	Qingdao Emergency Response Plan for Urban Rail Transit Operation Emergencies	July 2020	323.77	Eastern
P7	Tianjin Emergency Response Plan for Emergencies in Urban Rail Transit Operations	January 2022	293.14	Eastern
P8	Suzhou City Railway Transportation Operation Emergency Response Plan	August 2020	254.2	Eastern
P9	Ningbo Railway Transportation Operation Emergency Response Plan	January 2021	185.14	Eastern
P10	Wuxi City Urban Railway Transportation Operation Emergency Response Plan	April 2014	110.77	Eastern
P11	Emergency Response Plan for Emergencies in Xiamen Urban Rail Transit Operations (Revised in 2020)	November 2020	98.4	Eastern
P12	Jinan City Urban Railway Transportation Operation Emergency Response Plan	June 2019	84.1	Eastern
P13	Jiaxing City Urban Railway Transportation Operation Emergency Response Plan	December 2021	60.12	Eastern
P14	Changzhou City Railway Transportation Operation Emergency Response Plan	September 2019	54.03	Eastern
P15	Wenzhou City Railway Transportation Operation Emergency Response Plan	April 2021	53.51	Eastern
P16	Shaoxing City Urban Railway Transportation Operation Emergency Response Plan	August 2021	47.1	Eastern
P17	Wuhu City Urban Railway Transportation Operation Emergency Response Plan	September 2021	46.2	Eastern
P18	Nantong City Urban Railway Transportation Operation Emergency Response Plan	December 2021	39.18	Eastern
P19	Emergency Response Plan for Changsha Rail Transit Operation Emergencies	June 2021	209.66	Central
P20	Emergency Response Plan for Rail Transit Operation in Nanchang City	August 2016	128.45	Central
P21	Emergency Response Plan for Urban Rail Transit Operations in Taizhou City	April 2023	52.4	Central
P22	Luoyang City Railway Transportation Operation Emergency Response Plan	July 2021	42.46	Central
P23	Guiyang City Urban Railway Transportation Operation Emergency Response Plan	November 2017	74.37	Central
P24	Shijiazhuang Railway Transportation Operation Emergency Response Plan	July 2017	74.28	Central
P25	Emergency Response Plan for Urban Rail Transit Operations in Taiyuan City	November 2020	23.28	Central
P26	Chengdu Urban Railway Transportation Operation Emergency Response Plan	April 2022	652.04	Western
P27	Chongqing Urban Railway Transportation Operation Emergency Response Plan	January 2016	478.29	Western
P28	Xi’an City Railway Transportation Operation Emergency Response Plan for Emergencies (2022 Revision)	May 2022	298.42	Western
P29	Emergency Response Plan for Urban Rail Transit Operations in Kunming	January 2021	165.85	Western
P30	Emergency Response Plan for Urban Rail Transit Operation in Nanning City	January 2020	124.96	Western
P31	Wenshan Prefecture Emergency Response Plan for Urban Rail Transit Operation Emergencies	March 2023	13.4	Western
P32	Tianshui City Urban Rail Transit Operation Emergency Response Plan	July 2022	12.93	Western
P33	Shenyang City Railway Transportation Operation Emergency Response Plan	June 2021	216.68	Northeastern

Web sites of municipal governments in China, Peking University Fabulous Web site (https://www.pkulaw.com/).

### 3.2 Construction of the PMC-index model

**Fig 2:**
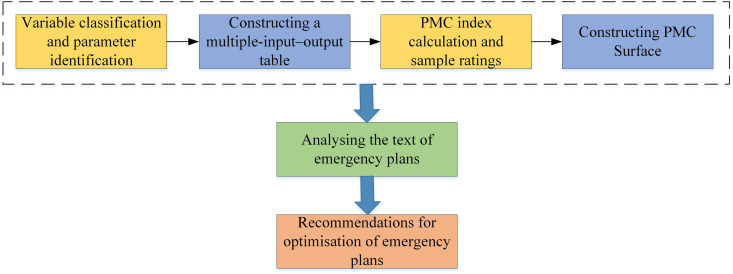
PMC index model evaluation process.

The PMC index model is a quantitative evaluation method that measures the effectiveness of policy making by constructing a comprehensive evaluation system with multiple indicators. This study primarily aims to assess the strengths, weaknesses, and consistency of URTOEPs policy texts through calculating PMC indexes based on various primary and secondary variables, as well as creating PMC surface graphs. As depicted in Fig 2, the logical framework for constructing the PMC index model and evaluating emergency plans includes: (1) variable selection and parameter identification; (2) development of a multi-input-output matrix; (3) computation of PMC indexes and sample rating; (4) visualization of PMC surfaces; (5) assessment of text quality and analysis of emergency plans; (6) recommendations for optimizing emergency plans.

#### 3.2.1 Variable classification and parameter identification.

In order to avoid subjective selection of evaluation criteria, this study employs the text analysis method to extract high-frequency terms from 33 emergency plans and conduct variable classification and extraction. This process aims to construct an evaluation system for public emergencies in urban rail transit operations. Firstly, the 33 emergency plans were imported into NVIVO11 software, and the top 300 high-frequency terms were extracted through file merging, word processing, and frequency statistics. Subsequently, distracting terms such as “unit,” “carry out,” “related to,” and “adopt” were eliminated while synonyms were merged (e.g., combining “municipal government,” “people’s government,” and “municipal party committee” into “government”; and merging “guide,” “direct,” and “command” into “command”). Ultimately, a total of 231 valid high-frequency terms were obtained.

The subjectivity associated with variable selection is mitigated in this paper through the application of textual analysis on the preprogramme, which involves the classification and integration of 231 effective high-frequency words. Subsequently, secondary variables are derived by synthesizing existing literature and the content of the emergency plan, thereby providing a foundational framework for primary variables. The categorization of high-frequency words and extraction table for variables are ultimately established (refer to Table 2). Through the categorization and extraction of high-frequency vocabulary, as well as integration with the Specification for the Preparation of Emergency Response Plans for Urban Railway Transport Operations (JT/T 1051–2016), and referencing relevant studies on emergency response plan evaluation, this study has ultimately developed a comprehensive system of indicators for evaluating emergency response plans in urban railway transport operations. The system encompasses 9 primary variables and 42 secondary variables (Table 3).

**Table 2:** Effective high-frequency vocabulary categorisation and variable extraction table.

Primary Variables	Corresponding Vocabulary
Plan basis	Criteria, provisions, bases, laws, rules, regulations, by law, principles, programmes, procedures
Applicability of plan	Application, scope, classification, types, earthquakes, strikes, failures, natural disasters, degree, level, grade, classification
Plan management	Preparation, Revision, Exercise, Publicity, Training, Education (Training), Publicity Department
Prevention preparation	Inspection (check, review), supervision, maintenance, periodicity, stockpiling, prevention, precaution, risk, secondary, preparedness, prediction (forecasting)
Monitoring and early warning	Monitoring, early warning, yellow, blue, red, orange, issuance, alerts
Command and coordination	Commander-in-Chief, Head, Organisation, Command (Guide, Direction, Command), Operational Unit, Expert, Expert Group, Coordination, Linkage, Sharing, Co-operation, Support, Assistance, Sub-ordination, Supervisor, Territoriality, Neighbourhood
Emergency response	Report (notify, record), escalate, report, dispose, rescue, repair, advance, alert, passenger flow, passengers, public, evacuate, channelise, transfer, response (answer, acknowledge, pay attention to), rescue, salvage, control, control, rescue, on-scene, adjust, society (group), service (help, supply), volunteer, news (report, message), broadcast, inform, telephone, public opinion, public opinion, Media, Elimination, End, Discharge, Termination
Recovery and reconstruction	Aftermath, Compensation, Relief, Donation, Fundraising, Restoration (Repair, Modification, Reconstruction), Ecology, Investigation (Analysis, Inspection), Summarisation, Assessment (Evaluation), Study, Research, Data
Safeguard measures	Personnel, members, teams, public security, armed police, firefighting, funds, finance bureau, materials, vehicles, medical, health, security, communications, technology, power supply, electricity

**Table 3:** Evaluation index system of emergency response plan for urban rail transport operation emergencies.

Primary Variables	Secondary variables	References
X1 Plan basis	X1:1 Compliance with the law	Specification for the Preparation of Emergency Response Plans for Emergencies in Urban Railway Transportation Operations and Text analysis
	X1:2 Purpose of preparation	
X2 Applicability of plan	X2:1 Scope of Application	Li et al. [38],Gao [39] and Text analysis
	X2:2 Incident classification	
	X2:3 Incident grading	
X3 Plan management	X3:1 Preparation and Revision	Xu [37], Specification for the Preparation of Emergency Response Plans for Emergencies in Urban Railway Transportation Operations and Text analysis
	X3:2 Program Exercises	
	X3:3 Sensitization and training	
X4 Prevention preparation	X4:1Daily supervision	Xu [37], Gao [39] and Text analysis
	X4:2Material stockpiling	
	X4:3Risk Assessment	
X5 Monitoring and early warning	X5:1 Information monitoring	Xu [37], Specification for the Preparation of Emergency Response Plans for Emergencies in Urban Railway Transportation Operations and Text analysis
	X5:2Determination of warning levels	
	X5:3 Warning information dissemination	
	X5:4 Warning lifted	
X6 Command and coordination	X6:1 Commander-in-chief/leading agency	Xu [37], Gao [39],Li et al. [38], Specification for the Preparation of Emergency Response Plans for Emergencies in Urban Railway Transportation Operations and Text analysis
	X6:2Urban rail transit operations emergency command/center	
	X6:3On-site command/lead agency	
	X6:4Operating units	
	X6:5Expert groups	
	X6:6Coordination linkage	
X7 Emergency response	X7:1Information reporting	Xu [37], Gao [39],Li et al. [38], Specification for the Preparation of Emergency Response Plans for Emergencies in Urban Railway Transportation Operations and Text analysis
	X7:2Response classification	
	X7:3Passenger evacuation	
	X7:4Passenger transfer	
	X7:5Repair and emergency response	
	X7:6Psychological Assistance	
	X7:7Social Assistance	
	X7:8Information dissemination	
	X7:9Public opinion guidance	
	X7:10 End of emergency	
X8 Recovery and reconstruction	X8:1 Aftercare	Xu [37],Gao [39] and Text analysis
	X8:2 Social Assistance	
	X8:3 Incident Investigation	
	X8:4 Disposal Assessment	
	X8:5 Learning and Research	
X9 Safeguard measures	X9:1 Team security	Xu [37], Specification for the Preparation of Emergency Response Plans for Emergencies in Urban Railway Transportation Operations and Text analysis
	X9:2 Financial security	
	X9:3 Equipment and material security	
	X9:4 Transportation Security	
	X9:5 Communication and Information Security	
	X9:6 Technology Security	

#### 3.2.2 Constructing a multiple-input–output table.

The construction of the assessment system necessitates the creation of a multi-input-output table and the establishment of variable parameters. The primary function of the multi-input-output table is to enhance the quantification of each sub-variable’s value, while also providing ample storage capacity for data required in individual variable calculations [26]. The parameter of the secondary variable is assigned as “1” if the text of the emergency plan to be evaluated meets the evaluation criteria according to the PMC index model variable settings and evaluation criteria for each secondary variable; otherwise, it is assigned as “0” [37]. The multiple inputs and outputs established in this paper are presented in Table 4.

**Table 4:** Table of multi-input-output.

Primary Variables	Secondary variables
X1	X1:1 X1:2
X2	X2:1 X2:2 X2:3
X3	X3:1 X3:2 X3:3
X4	X4:1 X4:2 X4:3
X5	X5:1 X5:2 X5:3 X5:4
X6	X6:1 X6:2 X6:3 X6:4 X6:5 X6:6
X7	X7:1 X7:2 X7:3 X7:4 X7:5 X7:6 X7:7 X7:8 X7:9 X7:10
X8	X8:1 X8:2 X8:3 X8:4 X8:5
X9	X9:1 X9:2 X9:3 X9:4 X9:5 X9:6

#### 3.2.3 PMC Index calculation and sample ratings.

The secondary variables were assigned binary values of “0” or “1” in order to ensure equal importance and weight. Specifically, equations (1) and (2) were used to calculate the specific values of these variables for each sample. Subsequently, equation (3) was applied to determine the values of the primary variables. Finally, the PMC index of each sample was calculated using equation (4).


X~N[0,1] (1)

X={XR:[0~1]}                                                                       (2)

$$\begin{array}{c} X_{t}\left(\sum {\mathrm{\backslash nolimits}}_{j=1}^{n}\frac{X_{t:j}}{T(X_{t:j})}\right),\;X_{t}\sim R[0\sim 1],\;t=1,2,3,4,5,6,7,8,9,10,\ldots ,\infty \end{array}$$ (3)

$$\begin{array}{cc} PMC= & X_{1}\left(\sum {\mathrm{\backslash nolimits}}_{\left(i=1\right)}^{2}\frac{X_{t:j}}{2}\right)+X_{2}\left(\sum {\mathrm{\backslash nolimits}}_{\left(i=1\right)}^{3}\frac{X_{t:j}}{3}\right)+X_{3}\left(\sum {\mathrm{\backslash nolimits}}_{\left(i=1\right)}^{3}\frac{X_{t:j}}{3}\right)+X_{4}\left(\sum {\mathrm{\backslash nolimits}}_{\left(i=1\right)}^{3}\frac{X_{t:j}}{3}\right)+X_{5}\left(\sum {\mathrm{\backslash nolimits}}_{\left(i=1\right)}^{3}\frac{X_{t:j}}{3}\right)\\ & +X_{6}\left(\sum {\mathrm{\backslash nolimits}}_{\left(i=1\right)}^{7}\frac{X_{t:j}}{7}\right)+X_{7}\left(\sum {\mathrm{\backslash nolimits}}_{\left(i=1\right)}^{8}\frac{X_{t:j}}{8}\right)+X_{8}\left(\sum {\mathrm{\backslash nolimits}}_{\left(i=1\right)}^{6}\frac{X_{t:j}}{6}\right)+X_{9}\left(\sum {\mathrm{\backslash nolimits}}_{\left(i=1\right)}^{9}\frac{X_{t:j}}{9}\right) \end{array}$$ (4)

The aforementioned steps lead to the acquisition of a comprehensive summary table illustrating PMC indexes for emergency response plans in urban rail transit operations, as presented in Table 5.

**Table 5:** Summary of PMC indices for URTOEPs.

Sample number	Values of the main variables									PMC
	X1	X2	X3	X4	X5	X6	X7	X8	X9	
P7	1.00	1.00	1.00	0.67	1.00	1.00	1.00	0.80	1.00	8.47
P12	1.00	0.67	1.00	1.00	1.00	1.00	1.00	0.80	1.00	8.47
P6	1.00	0.67	1.00	1.00	1.00	1.00	1.00	0.80	0.83	8.30
P8	1.00	0.67	1.00	0.67	1.00	1.00	0.90	1.00	1.00	8.23
P30	1.00	0.67	1.00	0.67	1.00	1.00	0.90	1.00	1.00	8.23
P1	1.00	0.67	1.00	1.00	1.00	1.00	0.90	0.60	1.00	8.17
P25	1.00	0.67	1.00	0.67	1.00	0.83	1.00	1.00	1.00	8.17
P19	1.00	0.67	1.00	0.67	1.00	1.00	1.00	0.80	1.00	8.13
P28	1.00	0.67	1.00	0.67	1.00	1.00	1.00	0.80	1.00	8.13
P3	1.00	0.67	1.00	1.00	0.75	1.00	1.00	0.60	1.00	8.02
P31	1.00	0.67	1.00	0.33	1.00	1.00	1.00	1.00	1.00	8.00
P17	1.00	0.67	1.00	0.33	1.00	1.00	0.90	1.00	1.00	7.90
P33	1.00	0.67	1.00	0.67	0.75	1.00	1.00	0.80	1.00	7.88
P14	1.00	1.00	1.00	0.33	1.00	0.83	0.90	0.80	1.00	7.87
P15	1.00	0.67	1.00	1.00	0.75	1.00	1.00	0.60	0.83	7.85
P24	1.00	0.67	1.00	0.67	1.00	1.00	0.90	0.60	1.00	7.83
P11	1.00	0.67	1.00	0.33	1.00	1.00	1.00	0.80	1.00	7.80
P13	1.00	0.67	1.00	0.33	1.00	1.00	1.00	0.80	1.00	7.80
P29	1.00	0.67	1.00	0.33	1.00	1.00	1.00	0.80	1.00	7.80
P20	1.00	1.00	1.00	0.67	0.50	1.00	0.80	1.00	0.83	7.80
P26	1.00	0.67	1.00	0.67	1.00	1.00	0.80	0.60	1.00	7.73
P21	1.00	0.67	1.00	0.67	0.75	1.00	1.00	0.80	0.83	7.72
P32	1.00	0.67	1.00	0.67	0.75	1.00	1.00	0.80	0.83	7.72
P4	1.00	1.00	1.00	0.33	1.00	1.00	0.70	0.80	0.83	7.67
P23	1.00	0.67	1.00	0.67	1.00	1.00	0.70	0.60	1.00	7.63
P18	1.00	1.00	1.00	0.67	0.75	0.83	0.90	0.60	0.83	7.58
P9	1.00	0.67	1.00	0.33	0.75	1.00	1.00	0.80	1.00	7.55
P5	1.00	0.67	1.00	0.33	1.00	1.00	0.90	0.60	1.00	7.50
P22	1.00	0.67	1.00	0.33	1.00	0.83	0.70	0.80	1.00	7.33
P2	1.00	0.67	1.00	0.67	1.00	0.83	0.70	0.60	0.83	7.30
P27	1.00	0.67	1.00	0.67	1.00	0.67	1.00	0.60	0.67	7.27
P16	1.00	0.67	0.67	0.33	1.00	1.00	0.90	0.60	1.00	7.17
P10	1.00	1.00	1.00	0.33	0.50	0.83	0.70	0.80	0.83	7.00
average value	1.00	0.73	0.99	0.60	0.92	0.96	0.92	0.77	0.94	7.82

**Fig 3:**
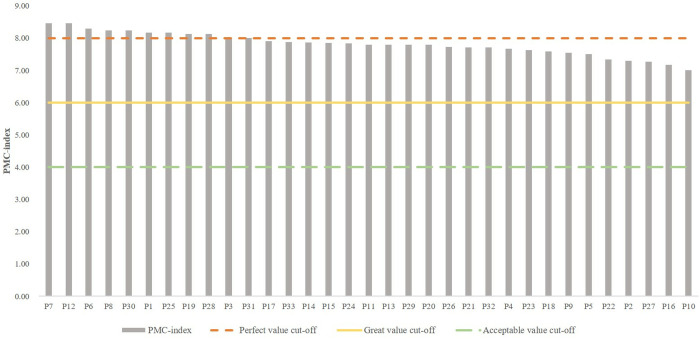
URTOEPs PMC index ranking and quality ratings.

After assessing the PMC index of the emergency preparedness text, we categorized the sample into different levels. In this study, following Mario Arturo Ruiz Estrada’s evaluation criteria [26], we refer to previous research and classify PMC into four grades (refer to Table 6): 9 > PMC > 8, Perfect consistency; 8 > PMC > 6, Great consistency; 6 > PMC > 4, Acceptable consistency; 4 > PMC > 0, Poor consistency. Ultimately, we obtain the text quality rating for emergency response plan in urban rail transit operation emergencies as depicted in Fig 3.

**Table 6:** Criteria for classifying the quality level of the text of the emergency plan.

PMC-Index	0 ~ 3.99	4 ~ 5.99	6 ~ 7.99	8 ~ 9
Evaluation	Poor consistency	Acceptable consistency	Great consistency	Perfect consistency

#### 3.2.4 Constructing PMC surface.

In order to visually represent the comprehensive results of the PMC indices for each sample, a pictorial representation in the form of a surface map can be utilized to demonstrate the strengths and weaknesses of emergency preparedness texts on a multidimensional coordinate space. The construction of this surface map involves utilizing scores from nine first-level variables to create a symmetrical surface. Therefore, it is necessary to establish the PMC matrix by incorporating the nine first-level variable indices into it and calculating matrix values based on Eq 5 before generating the PMC surface map.


$${PMC}_{surface}=\left\{\begin{array}{c} X_{1}\;X_{2}\;X_{3}\\ X_{4}\;X_{5}\;X_{6}\\ X_{7}\;X_{8}\;X_{9} \end{array}\right\}$$ (5)

**Fig 4:**
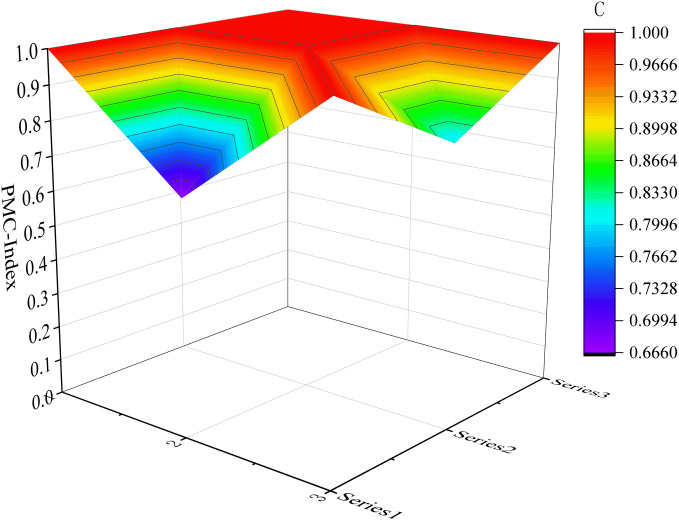
The PMC-Surface of P7.

**Fig 5:**
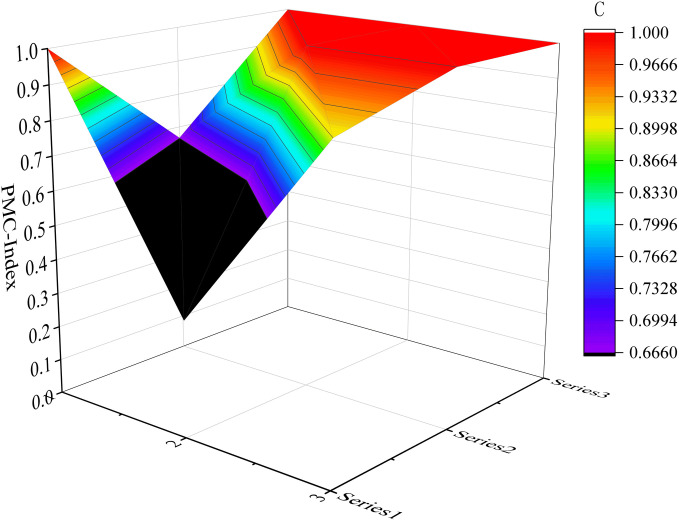
The PMC-Surface of P17.

**Fig 6:**
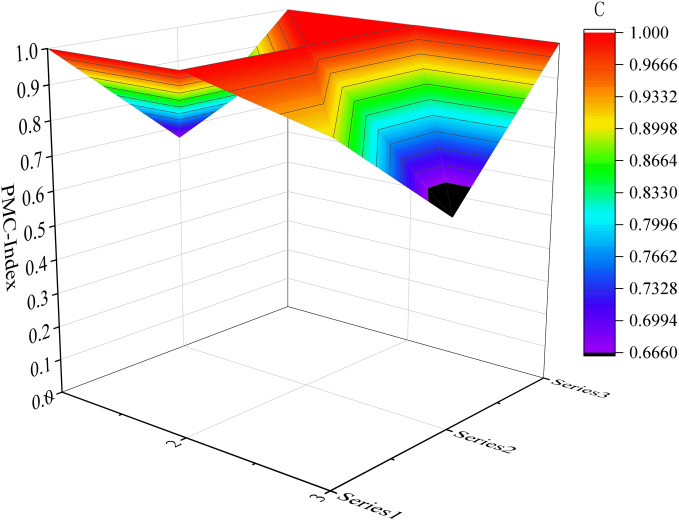
The PMC-Surface of P1.

**Fig 7:**
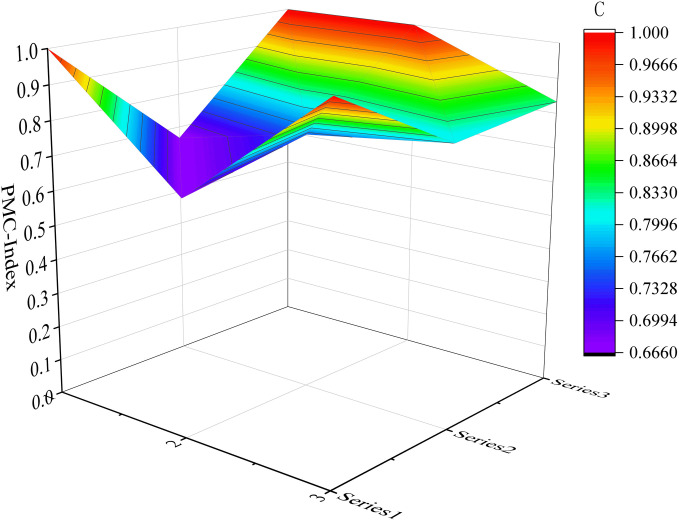
The PMC-Surface of P32.

**Fig 8:**
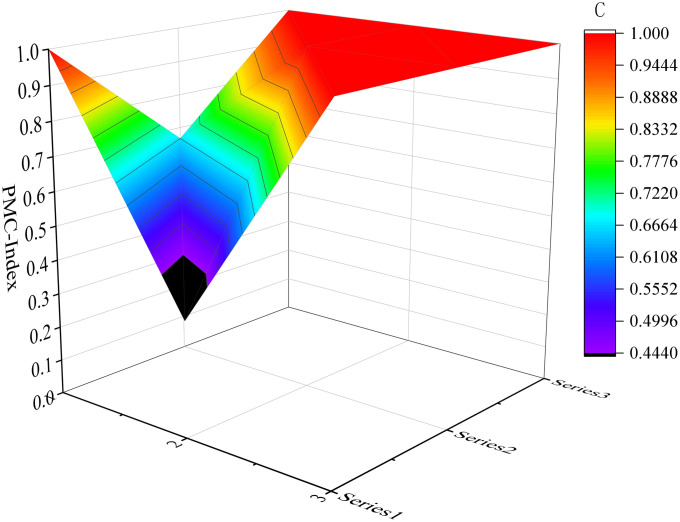
The PMC-Surface of P31.

**Fig 9:**
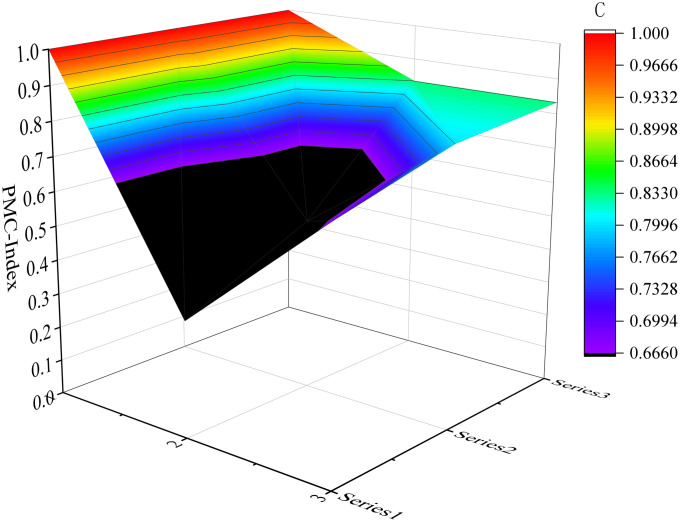
The PMC-Surface of P10.

Considering the limited space available, we have selected six samples as illustrative examples. These include the top-performing samples (P7 and P17) in the excellent and good grades, respectively, along with the moderately performing samples (P1 and P32), as well as the lowest-scoring samples (P31 and P10). Please refer to Figs 4–9 for more details. The horizontal sections in the surface plot represent the first-level variables X1 ~ X9, and the vertical axes 0 ~ 1 represent the corresponding PMC index values. Specifically, 1 ~ 3 of the horizontal coordinate represents the horizontal axis of the matrix, and series 1 ~ 3 of the vertical coordinate represents the vertical axis of the matrix, and the variables of the matrix can be represented by the coordinate system of the surface plot, such as the coordinate system of the variable X1 is (1, series 1), the variable X2 is (1, series 2), the variable X4 is (2, series 1), the variable X5 is (2, series 2), and the variable X7 is (3, series 1), and X8 is (3, series 2). From the surface plot, it can be seen that the different color blocks represent the high and low scores of the indicators, the red part represents the higher value of this coordinate, which means that the PMC index of this variable indicator is better for this scenario, and the black part is the worst, which means that the PMC index of this variable indicator is lower. The scores of each of the preplans can also be visualized through the undulations of the surface, with the raised portion representing a higher score and the concave portion representing a lower score. Thus, the comparison of the PMC surface plots shows the strengths and weaknesses of each preplan with respect to the different variable indicators.

## 4 Analysis of quantitative evaluation results

### 4.1 Overall characteristics of emergency plans

According to Table 5, overall, the mean value of PMC index of URTOEPs in Chinese cities is 7.83, indicating a good grade. Among them, a total of 11 URTOEPs achieved an excellent grade, while the remaining 22 URTOEPs were all rated as good. There were no URTOEPs with poor text quality observed. The mean values of the PMC indices for the nine main variables are ranked from high to low as follows: X1 (preparation basis) > X3 (plan management) > X6 (command and coordination) > X9 (safeguards) > X5 (monitoring and warning) > X7 (emergency response) > X8 (restoration and reconstruction) > X2 (application of plans) > X4 (preventive preparedness). The preparation basis stands out as the sole comprehensive indicator among the primary variables. The collected samples provide a detailed account of the purpose behind emergency plan preparation, along with the legal norms and technical standards upon which it is founded. This serves to demonstrate that China’s URTOEPs adhere to high levels of standardization, boasting ample scientific grounding and institutional benchmarks. In terms of indicator dimensions, Chinese URTOEPs exhibit three key aspects pertaining to text quality.

First and foremost, the plan management (X3) and command and coordination (X6) exhibit the highest mean values for the PMC index. In terms of plan management, 32 emergency plans received full scores, with only Shaoxing City scoring 0.67. This discrepancy arises from the absence of publicity and training components in Shaoxing City’s URTOEP. The text merely mentions that one responsibility of the Publicity Department of the Municipal Party Committee is to guide urban rail transit groups in safety promotion, without specifically addressing other educational and training tasks. This indicates that most URTOEPs have clearly stipulated provisions for plan preparation and revision, plan exercises, as well as publicity and training activities, thereby providing normative guidance for emergency plan management. The mean score for command and coordination indicator reaches 0.96; 26 emergency plans achieved perfect scores while outlining clear responsibilities and composition of municipal emergency organizations and command institutions. However, a majority of remaining emergency plans fail to explicitly explain operating units’ responsibilities within the organization and command system by employing vague expressions such as “relevant units” or “related units.”

Secondly, the mean values of restoration and reconstruction (X8) as well as the application of preplanning (X2) exhibit a low level. The primary objectives during the restoration and reconstruction phase encompass restoring the original functionality of urban rail transportation, ensuring an equitable allocation of relief resources, and conducting investigations into the root causes of emergencies. However, within this particular indicator for restoration and reconstruction, only 6 emergency plan texts achieved full marks, accounting for less than 20%. Determining and implementing aftercare programs, investigating emergencies thoroughly, and evaluating emergency response constitute pivotal tasks in the recovery and reconstruction stage. Amongst the secondary indicators, aftercare measures and incident investigations garnered commendable scores with all 33 emergency plan texts clearly articulating relevant details; whereas in terms of disposition assessment dimension, only one sample lacked corresponding expression. However, there is a varying degree of absence in the two indicators related to social assistance and learning and research. Specifically, 24 emergency plans fail to mention social assistance, while 13 lack relevant content regarding post-event learning and research. It can be observed that URTOEPs lack specific programs for social assistance and after-action learning research in the recovery and reconstruction phases, which require further improvement. The main reason for the low score of the first-level indicator of plan application (X2) is the failure to clearly categorize operational emergencies in the emergency plan texts. The limited availability of only six texts classifying emergencies in urban rail transit operations hinders the rational allocation of resources and the formulation of targeted response strategies by relevant departments, impeding efforts to reduce the likelihood and impact scope of operational emergencies.

Further, preventive preparedness (X4) exhibits the lowest mean PMC index value. Among its secondary indicators, the number of emergency plans lacking a risk assessment section in their content is the highest, reaching 27. By comprehensively analyzing and evaluating the risk factors impacting rail transit safety within the city and assessing the emergency response capabilities of transportation industry entities, we can obtain a comprehensive understanding of the primary risks and threats to rail transit safety in our city. This enables us to ensure prompt mitigation of key risks while also proactively formulating coordinated emergency response plans with other regions based on our own assessment results. Under the backdrop of expanding urban underground space development and utilization, as well as rapid and large-scale growth in rail transit, it is imperative for URTOEPs to enhance the scientific and effective risk assessment capabilities while objectively evaluating their own emergency response capacity. Furthermore, there are varying degrees of deficiencies in two secondary indicators contained within. For instance, six plans fail to mention the specifics of daily supervision work pertaining to URTOEPs’ safety, while seven emergency plans inadequately address material stockpiles with limited relevant expressions found within the texts.

### 4.2 Comparison of different levels of emergency plans

**Fig 10:**
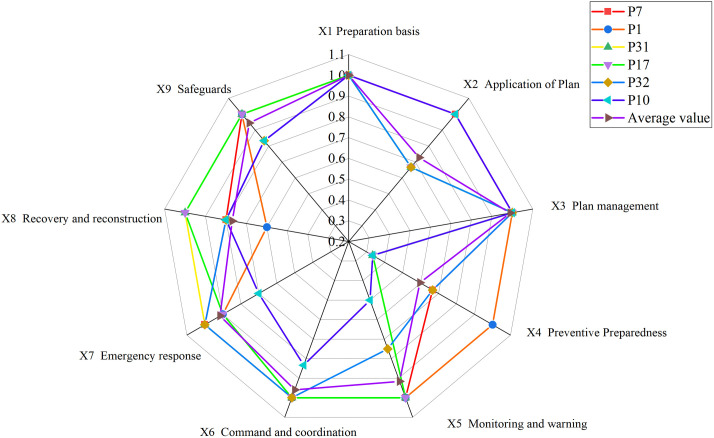
PMC surface map of URTOEPs.

In order to provide a more intuitive representation of the scores for different levels of representative samples, this paper utilizes Dybla diagrams as supplementary tools to surface diagrams in visualizing the indicators’ scores pertaining to the emergency plan text. As depicted in Fig 10, the visualization showcases both the scores and changes observed in six selected representative samples of emergency plans.

#### 4.2.1 Evaluation of URTOEPs with perfect ratings.

One third of the 33 texts in the sample obtained an excellent rating on the PMC index measure. These 11 texts achieved a perfect score of 100% on X1 preparation basis and X3 plan management; while 10 texts attained flawless scores on X5, X6, and X9. However, there were more deficiencies observed in indexes X2, X4, and X8. This observation suggests that municipal-level URTOEPs in China have well-defined specifications for monitoring and early warning systems, command and coordination mechanisms, emergency response protocols, as well as safeguards for managing operational emergencies. It reflects the standardized actions undertaken by municipal governments to effectively respond to emergencies and fulfill their primary role in emergency response.

Among them, P7 has the highest PMC value, corresponding to the Emergency Response Plan for Urban Railway Transportation Operations in Tianjin. All 7 indicators in the main index receive full marks, indicating that the emergency plan is well-designed, scientifically sound, and comprehensive. It is worth noting that compared to other plans, this emergency plan provides more detailed response grading and measures. It includes specific response measures for different levels of operational emergencies such as information reporting, on-site command, organization and coordination, and emergency disposal. The flow chart also visually illustrates the links of urban rail transit operations’ emergency response. Additionally, it is also emphasized that the response level can be dynamically adjusted based on the incident to prevent inadequate or excessive response. Furthermore, the need for expanding the response has been adequately explained. However, there are still areas in the text that require improvement: firstly, prevention and preparation (X4) fails to identify and summarize potential risks associated with urban rail transit operation in the city; secondly, recovery and reconstruction (X8) lacks sufficient and specific description of social assistance, which should be tailored to Tianjin’s actual situation for an enhanced plan. Therefore, it is recommended to enhance the main variables of P7 as follows: X4-X8.

The PMC value of P1 is ranked sixth, corresponding to the Emergency Response Plan for Emergencies in Shanghai Railway Transportation Operations. The plan is relatively comprehensive overall, with all 6 level 1 indicators scoring full marks, and it contains numerous commendable aspects. For instance, the plan includes a dedicated chapter on risk assessment that adopts a “people - machine - environment - management” perspective, focusing on analyzing the four major risk factors that impact the safety of Shanghai rail transit operations. In comparison to other emergency response plans which merely touch upon or even omit this aspect entirely, this plan anticipates and evaluates potential threats to urban rail transit operation in the city more effectively. This provides decision makers with greater insight into the risk situation and facilitates investigation into key hidden dangers. However, based on the surface diagram of P1, it can be observed that the plan exhibits a slight concave state overall. This is primarily attributed to the relatively low scores of the plan application (X2), emergency response (X7), and restoration and reconstruction (X8) components, which are all below their respective mean values as depicted in Fig 10. The plan application (X2) fails to adequately address urban rail transit operation emergencies, while the emergency response (X7) lacks proper organization and guidance for social rescue forces. Additionally, the recovery and reconstruction aspect (X8) suffers from a lack of diverse executive bodies, insufficient description of social assistance content, and an absence of post-event study and research. In light of these findings, it is recommended to optimize P1 by prioritizing variables in the following order: X8-X2-X7.

The PMC value of P31 is ranked eleventh, corresponding to the Emergency Response Plan for Emergencies in Urban Rail Transportation Operations in Wenshan State. From the surface diagram of P31, it can be clearly observed that there are two surfaces exhibiting significant concavity and black areas. When combined with the Dybra diagram (Fig 10), it becomes evident that the text of the Wenshan State Emergency Response Plan demonstrates a relatively low level of completeness in terms of plan application (X2) and preventive preparedness (X4). Moreover, these values are lower than their respective averages, particularly highlighting the urgent need for optimization and improvement in preventive preparedness (X4). Therefore, it is recommended to prioritize optimization efforts on key variables such as X4-X2.

#### 4.2.2 Evaluation of URTOEPs with great ratings.

Twenty-two out of the URTOEPs sample achieved a good rating, constituting two-thirds of the evaluated sample size. However, it is worth noting that the scores for plan application (X2), preventive preparation (X4), emergency response (X7), and recovery and reconstruction (X8) indicators are relatively low and require further improvement.

Regarding the implementation of the plan (X2), only 5 out of 22 good grade samples mentioned the categorization of urban rail transit operation emergencies, which generally classify them into four categories: natural disasters (such as earthquakes, floods, meteorological disasters), social security incidents (including terrorist attacks, criminal cases, sudden surges in passenger flow), public health crises (such as infectious disease outbreaks, biochemical and toxic gas contamination, radioactive exposure), and accidental catastrophes (like train collisions, derailments, facility and equipment failures). On the dimension of preventive preparedness (X4), there are only two good grade texts that incorporate risk assessment. One is P15, titled “Emergency Response Plan for Emergencies in Wenzhou City Urban Rail Transit Operations,” which elucidates the risks associated with urban rail transit operations in Wenzhou City and provides a concise overview of the emergency resources available for the city’s urban rail transit operations, including the emergency response plan, emergency response team, and emergency response materials. The second text is P33, titled “Emergency Response Plan for Urban Rail Transit Operation in Shenyang City,” primarily encompassing relevant requirements for risk identification and assessment of emergency response capacity. For Emergency Response (X7), 8 good grade texts do not specifically address psychological assistance during the process of emergency response. The Recovery and Reconstruction (X8) plans lack specification regarding social assistance in 19 texts. Instead, most of the plans use terms like “post-disaster work plan” and “insurance agency for claim settlement,” without clearly outlining the division of responsibilities among organizational departments.

The PMC value of P17 is 7.90, ranking first in the good grade category, corresponding to the Emergency Response Plan for Urban Railway Transportation Operation in Wuhu City. The curved surface diagram of P17 exhibits a significant degree of concavity in the preventive preparation part (X4). When combined with the Debra diagram, it becomes evident that the scores for plan application (X2) and emergency response (X7) are also below the average score for this index. Specifically, there are three areas that require optimization: firstly, emergency classification; secondly, material stockpiling and risk assessment for urban rail transit within the city; and thirdly, inclusion of content related to psychological assistance in the emergency response section. Therefore. It is suggested that the idea of optimizing the main variables of P17 is: X4-X2-X7.

The PMC value of P32 is 7.72, which aligns with the Emergency Response Plan for Emergencies in Urban Rail Transportation Operations in Tianshui City. This plan exhibits a relatively comprehensive approach towards preparation basis, plan management, command and coordination, as well as emergency response. However, there are certain aspects that require further elaboration within the application of the plan (X2), preventive preparation (X4), monitoring and warning (X5), recovery and reconstruction (X8), and safeguard measures (X9). It is suggested that the optimization idea for the main variables of P32 is: X4-X2-X5-X8-X9.

The PMC value of P10 is 7.00, ranking last and corresponding to the Emergency Response Plan for Emergencies in Urban Rail Transportation Operations in Wuxi City, which was promulgated in April 2014. In this plan, X1 Preparation Basis, X2 Application of the plan, and X3 Management of the plan have achieved full marks; however, other sections lack relevant provisions to varying degrees. Additionally, the surface diagram of P10 indicates areas that require improvement within the plan. Despite stating a requirement for periodic revision and improvement based on actual circumstances, it is worth noting that the plan has not been revised since its initial issuance. Therefore, it is recommended that P10 clarify both the revision cycle and main body of the plan while evaluating its implementation periodically. The main variables are optimized along the following lines: X4-X5-X7-X6-X8-X9.

## 5 Discussion

The empirical analysis of 33 URTOEPs reveals the state of emergency planning in Chinese local governments and offers a comprehensive examination of the challenges and policy recommendations for emergency planning in key areas of urban safety management.

### 5.1 Main findings

(1) The overall quality of China’s URTOEPs is commendable, although there is variation in the quality of individual plans. At an aggregate level, the 33 URTOEPs have achieved an average score of 7.82, indicating a high standard. Consequently, it can be inferred that the current municipal-level URTOEPs in China possess a certain degree of operational effectiveness in responding to emergencies and provide effective guidance at lower administrative levels. Regarding the structural integrity of these plans, two-thirds of them are rated as satisfactory, with the highest PMC index value recorded at 8.47 and the lowest at 7.00; thus suggesting insignificant inter-governmental disparities in terms of text quality within emergency plans. After analyzing the samples, it is evident that although each sample exhibits varying degrees of deficiencies in different indices on an individual basis, resulting in variations in text quality, there exists a high level of “transplantation” regarding the framework structure and content elements of emergency response plans for rail transit operation across cities. However, these plans fail to adequately emphasize the unique characteristics associated with risks within urban underground spaces.
(2) The texts of emergency plans exhibit greater comprehensiveness in terms of their foundation for preparation, plan management, and command and coordination. However, the quality of these texts is comparatively lower when it comes to plan application, prevention and preparedness, as well as recovery and reconstruction efforts. In regard to secondary indicators, the primary reason behind this diminished quality lies in the insufficient content pertaining to incident categorization, risk assessment, social assistance, and study and research. Firstly, the emergency plan lacks a classification system for potential emergencies that may arise during operational processes; consequently impeding effective implementation. Secondly, the construction of urban rail transit is well underway and its operational risks have gradually exhibited characteristics of coupling, cascading, and secondary disasters. However, analysis results indicate that many plans fail to integrate the operating environment of urban rail transit with potential risks and do not conduct a reasonable assessment of their own emergency response capabilities. Furthermore, social assistance and research are crucial components for recovery and reconstruction; however, few plans provide detailed requirements for these two aspects. Consequently, the policy effectiveness of emergency preparedness plans is limited.
(3) The timely revision and updating of certain URTOEPs in terms of time series were not adequately addressed. In accordance with China’s recently enacted Measures for the Administration of Emergency Response Plans for Emergencies, it is stipulated that the emergency response plans of local people’s governments at or above the county level and their relevant departments should undergo evaluation once every three years as a general principle. The sample includes 8 emergency plans introduced between 2014 and 2019, which are currently in effect without undergoing any revisions or improvements during the process. Moreover, all of these 8 emergency plans have received commendable PMC indexes. However, due to the emergence of a risk society and the rapid development of urban rail transit in China, there is an increased demand for enhanced functionality and comprehensiveness in emergency plans. Therefore, it is imperative to urgently address the existing situation of “no revision for years”.

### 5.2 Theoretical discussion

In recent years, there has been a significant surge in the construction of urban rail transit systems, with numerous countries and regions allocating substantial resources towards the development of this highly efficient mode of public transportation. Urban rail transit has emerged as the fundamental backbone of urban public transportation systems and plays a pivotal role in facilitating sustainable urban development [40]. Due to its extensive passenger attraction range and significant influence, the potential for mass casualties and injuries in emergency situations is considerably high. The emergency plan serves as a guiding document for effective emergency management. As an integral component of the comprehensive urban rail transit operation emergencies plan system, the municipal-level emergency plan must consider specific hazard characteristics in addition to incorporating special requirements and provisions from national and provincial level plans. Therefore, distinct from previous studies that discuss policy perspectives on emergency plan preparation and policy revision agendas, this study focuses on quantitatively exploring the technical aspects of specifying the fundamental contents of municipal-level emergency plans. By complementing existing research on evaluation indicators for emergency plans, this study also provides data support for optimizing the development and dynamic management of specialized emergency plans. However, it is important to acknowledge that this study primarily focuses on the internal consistency and structural integrity of emergency plans, and as such, the assessment results may not accurately reflect the practical application’s effects and challenges of these plans. The quality evaluation of emergency response plans encompasses various aspects including plan completeness, effectiveness, adaptability, feasibility, and timeliness; all of which cannot be fully captured by the dimensions outlined in the PMC model.

Secondly, the contingency plan is not merely a unit or organization’s strategy for constructing an emergency response system or an implementation plan for developing emergency response capacity. Instead, it serves as a proficient response program that takes into account the current risk situation and emergency response capabilities, enabling the identification, mobilization, and appropriate utilization of existing emergency response resources. The fundamental objective behind its formulation lies in transforming concerns regarding potential adverse consequences of emergencies into proactive thinking and planning, with the ultimate aim of achieving effective responses to and management of such situations. Thus, the key to constructing an effective emergency planning system lies in leveraging the efficacy of the emergency plan itself, rather than using it as a post-event accountability criterion. Moreover, maximizing the effectiveness of the emergency plan hinges on an organization’s adept utilization of the knowledge embedded within it to enhance its practical adaptability [8].

In addition, the emergency response plan is not a panacea. It is merely a pre-established program of action that cannot address every possible scenario and should not be solely relied upon to resolve all issues during an emergency response. Furthermore, it would be incorrect to attribute the ineffectiveness of an emergency response solely to the poor operation or lack of practicality of the plan. Emergency response plans should not strive for complete inclusivity; attempting to cover every conceivable situation or solve every minute detail will only impede and restrict those responsible for its implementation, ultimately hindering effective emergency response.

### 5.3 Practical implications

(1) The shift from “template replication” to “localization” is necessary. There is a need to transition from the traditional “top-down” template orientation to a more focused and comprehensive “bottom-up” risk orientation. Among these changes, security risk assessment and emergency response capacity assessment serve as the fundamental basis for emergency plan preparation.

The first step involves establishing the preliminary organization of various departments to conduct a comprehensive safety risk assessment for urban rail transit. This assessment will focus on the closed field, environmental complexity, and risk interconnections associated with urban rail transit. Following the logical sequence of “risk identification - risk analysis - risk evaluation,” it is essential to thoroughly evaluate potential risks that may occur during urban rail transit operations. This includes analyzing the likelihood and progression of such risks, as well as their potential impact on casualties, property damage, environmental hazards, and social consequences. Ultimately, a comprehensive, targeted and regularly updated safety risk assessment report will be completed for each urban rail transit system in every city. The emergency plan preparation team will then integrate and summarize the report to ensure standardization, scientificity and coordination of the emergency plan’s “first kilometer”. Additionally, each city will conduct an all-encompassing assessment of their operational emergency response capabilities based on the current situation of their urban rail transit operations. This includes evaluating their emergency plan systems, emergency rescue teams, emergency supplies as well as the effectiveness of their drills and training.

(2) The content of the plan should be standardized to transition from a “stand-alone” approach to a more “synergistic” one. The text of the emergency response plan should be enhanced and expanded in order to address the current lack of content pertaining to social assistance, as well as learning and research within the context of recovery and reconstruction.

In addition to regulating the government’s conduct during the recovery and reconstruction process, it is imperative for the plan to foster innovative approaches towards reconstruction assistance and actively encourage social engagement. The formulation of an emergency plan that mobilizes societal forces in humanitarian aid, psychological counseling, legal support, and other post-disaster relief programs can effectively integrate resources, transcend a sole reliance on governmental departments for system construction orientation, and establish a multi-stakeholder recovery and co-construction model with the government at its core. Particularly in the face of major disasters, revitalizing affected areas necessitates not only robust governmental backing but also contributions from all sectors of society.

(3) The management of updating the plans has shifted from “merely storing them” to “actively revising”; the management and updating of URTOEPs is itself a systematic and intricate process, which should prioritize timeliness and effectiveness. Therefore, on one hand, our focus should be on timely revisions. Apart from following the revision cycle outlined in regulations, accident reminders and policy adjustments also present suitable opportunities for revising emergency plans for urban rail transportation emergencies, as they directly impact the plan’s timeliness.

On the other hand, following major emergencies and relevant policy updates, it is imperative to thoroughly review and address any gaps in the plan’s text based on deficiencies identified throughout various stages of emergency management. This will enhance the plan’s quality through targeted and precise optimization strategies. Additionally, regular assessments are conducted to evaluate the plan’s effectiveness. Firstly, qualified and experienced third-party organizations are selected to ensure accurate and reliable evaluation results. Secondly, a comprehensive file management system is established for categorizing and archiving assessment outcomes as well as improvement measures, thereby establishing a continuous improvement mechanism.

### 5.4 Research outlook

Although this study provides data support for optimizing the construction of special emergency response plans in the field of urban rail transportation, there are still some research deficiencies. (1) This paper primarily selects a sample of URTOEPs at the local level, which limits the scope of the study. Subsequent studies can further expand the collection level and scope of policy text analysis. Additionally, apart from municipal-level analysis, it is possible to quantify and visualize the inter-organizational network structure and relationship patterns among national-provincial-municipal-URTOEPs from a vertical perspective. (2) There is room for improvement in the quantitative evaluation system constructed in this paper as its indicators lack dimensionality and comprehensiveness. Researchers can define standardized variables based on fundamental characteristics of emergency plans while also defining non-standardized variables specific to their research problem’s characteristics. This approach will aid in developing a comprehensive and targeted quantitative evaluation system. (3) Although the quantitative evaluation system constructed in this study realizes the structured assessment of the text quality of emergency plans through the PMC index model, it is undeniable that there are still limitations in measuring the effectiveness of the application of emergency plans, with the index dimensions focusing on the normative and completeness analysis of the text structure and the lack of in-depth portrayal of the dynamic implementation of the plan and the complex scenarios of the emergency management of urban rail transit. In the future, we can integrate multi-source methods to make up for the blind spots of the quantitative model, such as selecting cities with large differences in PMC scores to conduct comparative studies, and exploring the specific implementation dilemmas and optimization directions of the plan text through participatory observation or process tracking methods.

## Supporting information

S1 Table Multi-input-output table for 33 URTOEPs. (DOCX)
